# 临床试验不良事件监管在可手术III期非小细胞肺癌新辅助治疗中的作用

**DOI:** 10.3779/j.issn.1009-3419.2023.102.23

**Published:** 2023-06-20

**Authors:** Yun ZHANG, Shuang ZHOU, Wentao TAO, Rong LI

**Affiliations:** ^1^200030 上海，上海市胸科医院（上海交通大学附属胸科医院）临床研究中心; ^1^Shanghai Chest Hospital, Clinical Research Center, Chest Hospital Affiliated to Shanghai Jiaotong University, Shanghai 200030, China; ^2^上海交通大学（中国医院发展研究院）研究所; ^2^Research Institute of China Hospital Development Research Institute, Shanghai Jiao Tong University, Shanghai 200020, China

**Keywords:** 新辅助治疗, 肺肿瘤, 不良事件, Neoadjuvant therapy, Lung neoplasms, Adverse events

## Abstract

**背景与目的:**

程序性细胞死亡受体1（programmed cell death protein 1, PD-1）抑制剂联合含铂双药化疗是用于可手术III期非小细胞肺癌（non-small cell lung cancer, NSCLC）的新辅助治疗方法，相关药物临床试验的质量保证对试验结果起到至关重要的作用。本研究旨在探讨监管不良事件（adverse events, AEs）对降低患者治疗相关AEs的影响。

**方法:**

前瞻性收集2020年7月-2021年10月上海市胸科医院收治的NSCLC患者66例，均接受卡瑞利珠单抗联合多西他赛和顺铂新辅助治疗3个周期，新辅助治疗完成后4周-6周内接受手术；术后30 d内接受1个周期的术后辅助治疗，术后辅助治疗完成3周后进入卡瑞利珠单抗巩固治疗阶段，总计13个周期。采用生命质量测定量表（quality of life-C30, QoL-C30）测定患者生活质量，并监测AEs的发生情况。

**结果:**

总体安全性良好，66例患者共发生300次AEs，其中1级-2级AEs 282人次，3级-4级AEs 18人次。最常见的与PD-1抗体相关的3级-4级AEs为6次（9.1%）。监管新辅助治疗情况可使患者QOL-C30评分下降（P<0.05），生活质量提高。

**结论:**

卡瑞利珠单抗联合多西他赛和顺铂用于可手术III期NSCLC的新辅助治疗，通过AEs的观察与管控，可以及时采取处理措施，减少并发症的进一步发生，保证患者安全，确保临床试验数据的真实、科学、可靠。

## Abstract (en)

**Background and objective:**

Programmed cell death protein 1 (PD-1) combined with platinum containing dual drug chemotherapy is a new adjuvant treatment option for operable stage III non-small cell lung cancer (NSCLC), and the quality assurance of clinical trials of related drugs plays a crucial role in the results of the clinical trials. This study aims to explore the impact of adverse events (AEs) supervision on reducing treatment-related AEs in patients.

**Methods:**

66 NSCLC patients admitted to Shanghai Chest Hospital from July 2020 to October 2021 were prospectively collected. All the patients received 3 cycles of neoadjuvant treatment of Camrelizumab in combination with Docetaxel and Cisplatin. 4 weeks-6 weeks after neoadjuvant therapy, the patients accepted surgical treatment. One cycle of postoperative adjuvant treatment was given within 30 days after surgery, and 3 weeks after the completion of postoperative adjuvant treatment, Camrelizumab consolidation treatment was intiated, with a total of 13 cycles. The quality of life-C30 (QoL-C30) was used to measure patients' quality of life and the occurrence of AEs was monitored.

**Results:**

The overall safety is good, with a total of 300 AEs occurring in 66 patients, including 282 cases of grade 1-2 AEs and 18 cases of grades 3-4 AEs. The most common grades 3-4 AEs associated with PD-1 antibodies occurred in 6 cases (9.1%). Neoadjuvant therapy supervision can lead to a decrease in patients' QOL-C30 scores (P<0.05) and an improvement in their quality of life.

**Conclusion:**

Camrelizumab combined with Docetaxel and Cisplatin can be used as a new adjuvant treatment for operable stage III NSCLC. Through the observation and control of AEs, treatment measures can be taken in time to reduce further complications, ensure patient’ safety, and ensure the authenticity, scientificity and reliability of clinical trial data.

2020年统计数据^[1]^显示，在全球范围内肺癌发病率位居第二、死亡率高居第一，成为影响人类生命健康的公共卫生疾病。研究^[2]^显示，随着医疗卫生技术的进步及免疫治疗、靶向治疗等的发展，肺癌治疗水平不断提升，患者死亡率呈现下降趋势。手术、化疗和放疗是临床治疗肺癌的三大常见手段，其可改善患者病情、延长患者生存期，但患者仍面临复发转移的风险。目前，临床相关指南推荐将靶向药物、免疫药物应用于非小细胞肺癌（non-small cell lung cancer, NSCLC）治疗中。早在20世纪即有临床医生提出新辅助化疗这一概念，其强调在正式治疗前给予患者全身化疗，可缩小肿瘤体积，降低术前分期，进而降低手术难度，提高完整切除率^[3]^。目前关于NSCLC的化疗方案较多，其中以铂类为基础的新辅助化疗应用最为普遍，成为NSCLC患者新辅助化疗的首选^[4⇓-6]^。有研究^[7]^报道新辅助化疗方案在提升患者5年生存率方面有着显著疗效，但手术可切除肺癌患者生存率仍仅为50%左右，难以进一步提高。新辅助化疗联合免疫治疗在临床的应用为NSCLC患者带来了新的希望，NSCLC荟萃分析协作组^[8]^对15项NSCLC随机对照试验进行汇总，结果显示新辅助化疗联合程序性细胞死亡受体1（programmed cell death protein 1, PD-1）抑制剂可将患者相对死亡风险控制在较低范围，并能进一步提高临床疗效。但是新辅助化疗易引起机体不同程度的损害，患者会出现一系列不良事件（adverse events, AEs）。AEs指在临床试验过程中，受试者接受某种药物治疗后所产生的不良医学事件，但该事件并非与治疗绝对相关，如骨髓抑制、胃肠反应、肝肾功能损伤、高血压、免疫性相关器官损伤、脱发等，导致患者治疗依从性下降，临床疗效降低，医患纠纷增加，严重AEs（severe AEs, SAEs）甚至会导致患者死亡。AEs的及时上报关系着临床试验药物的监测结果，甚至直接关系到患者的安全问题。密切观察患者AEs发生情况并掌握各项AEs处理要点等，是降低临床风险、改善患者预后的有效举措^[9,10]^。如何监管新辅助化疗过程中的AEs发生情况，成为肿瘤科医护人员不可忽视的工作之一。

本研究选取于我院收治的接受PD-1抑制剂联合含铂双药化疗可手术的IIIA期-IIIB期NSCLC患者，分析PD-1抑制剂联合化疗的应用疗效与安全性，探讨监管AEs对降低患者治疗相关AEs的影响。

## 1 资料与方法

### 1.1 研究对象

前瞻性收集2020年7月-2021年10月上海市胸科医院收治的NSCLC患者66例，其中腺癌21例，鳞癌45例；男性60例，女性6例；平均年龄（62.0±6.7）岁；临床分期：IIIA期42例，IIIB期24例；美国东部肿瘤协作组（Eastern Cooperative Oncology Group, ECOG）体能状态评分：0分27例，1分39例；手术方式：肺癌根治术20例，全肺切除术2例，支气管袖式成型肺叶切除术35例，肺段楔形切除术9例；是否吸烟：是21例，否45例。患者一般资料见表1。

**表1:** 患者一般资料（n=66）

Index	Data
Age (Mean±SD, yr)	62.0±6.7
Gender	
Male	60 (90.9%)
Female	6 (9.1%)
Pathological type	
Adenocarcinoma	21 (31.8%)
Squamous cell carcinoma	45 (68.2%)
Clinical stage	
IIIA	42 (63.6%)
IIIB	24 (36.4%)
ECOG performance status score	
0	27 (40.9%)
1	39 (59.1%)
Operation method	
Radical resection	20 (30.3%)
Pneumonectomy	2 (3.0%)
Bronchial sleeve shaped lobectomy	35 (5.3%)
Wedge resection of lung segment	9 (13.6%)
Smoking history	
Yes	21 (31.8%)
No	45 (68.2%)

EOCG: Eastern Cooperative Oncology Group.

纳入标准：（1）病理诊断明确，为IIIA期-IIIB期NSCLC患者；（2）均为首次确诊，1个月内未行任何抗肿瘤治疗；（3）年龄18岁-75岁；（4）患者和家属充分知晓并签署同意书；（5）病历资料完整。排除标准：（1）药物过敏史者；（2）合并二次原发性肿瘤患者；（3）严重内科基础疾病或远处转移者；（4）哺乳期或妊娠期女性；（5）精神疾病不能配合治疗者。本研究经过医院伦理委员会审核通过。

### 1.2 研究方法

新辅助治疗方案：注射用卡瑞利珠单抗（规格：200 mg/瓶，苏州盛迪亚生物医药有限公司，国药准字：S20190027），200 mg/次，d1；联合多西他赛注射液（规格：80 mg/2 mL，扬子江药业集团有限公司，国药准字：H20174009）70 mg/m^2^-75 mg/m^2^，d1；注射用顺铂（规格：10 mg/瓶，德州德药制药有限公司，国药准字：H37020525）20 mg/m^2^，d1-d5，21 d为1个周期；完成新辅助治疗3个周期后，若患者符合手术指征，可在4周-6周内实施手术行手术治疗，手术方式分别为肺癌根治术、支气管袖式成型肺叶切除术、全肺切除术和肺段楔形切除术。术后30 d内接受1个周期的卡瑞利珠单抗联合多西他赛和顺铂术后辅助治疗，3周后进入卡瑞利珠单抗巩固治疗阶段，总计13个周期。治疗前及治疗3个周期后分别进行计算机断层扫描（computed tomography, CT）诊断检查，明确肿瘤病灶情况，记录肿瘤体积、形态变化等指标。

### 1.3 AEs严重程度及判断标准

包括AEs的严重性、严重度、预期性和相关性判断，严重性主要依据该事件是否为SAEs；严重度是针对AEs发生后所造成的后果严重程度进行的评估与分级，世界卫生组织（World Health Organization, WHO）对此有明确的分级；以美国国家癌症研究所常见不良反应事件评价标准（National Cancer Institute-Common Terminology Criteria Adverse Events, NCI-CTCAE）5.0关于药物的AEs为标准，并对AEs发生前后的状况以及对任何潜在原因的评价，确定AEs的相关性。若明确AEs为SAEs或可疑非预期的严重不良反应（suspected unexpected serious adverse reaction, SUSAR），应予以上报，以《药物临床试验期间快速报告的标准和程序》为标准，按照相关规定完成上报^[11]^。

### 1.4 生活质量

通过欧洲癌症治疗研究组织（European Organisation for Research and Treatment of Cancer, EORTC）生命质量测定量表（quality of life-C30, QoL-C30）调查肺癌患者生活质量问卷^[12⇓-14]^，了解患者健康及日常生活状态，共有30个问题。前28个问题以没有、有点、相当、非常相对应的1分-4分分值评价患者的日常状态，第29、30题分为7个等级，以1分-7分值，评价患者过去1周的健康情况和生活质量，得分越低表示生活质量越高。比较患者术前和术后问卷调查结果，评价患者生活质量评分。

### 1.5 统计学处理

使用SPSS 23.0进行统计数据分析，计数资料用率或者百分比（%）表示。计量资料采用均数±标准差（Mean±SD）表示，组间比较采用t检验。以P<0.05表示差异具有统计学意义。

## 2 结果

### 2.1 AEs及SAEs发生率

#### 2.1.1 免疫相关AEs

新辅助治疗期间免疫相关AEs共发生144次，其中1级-2级AEs为132次，3级-4级AEs为12次，SAEs为6次。围手术期和术后均未发生SAEs，新辅助治疗后手术治疗是安全可耐受的。最常见的与PD-1抑制剂相关的AEs为骨髓抑制，包括贫血27例（40.9%）、血小板减少18例（27.3%）、中性粒细胞减少12例（18.2%）、白细胞减少6例（9.1%）等。共6例发生SAEs，其中3例为肝功能异常4级，3例为中性粒细胞减少4级。4级患者永久性终止免疫治疗，并积极对症治疗后好转（表2）。

**表2:** 免疫相关不良事件

Adverse events	CTCAE 5.0 grade			Total
	Grade 1-2	Grade 3	Grade 4	
Anemia	27 (40.9%)	0 (0.0%)	0 (0.0%)	27 (40.9%)
Thrombocytopenia	18 (27.3%)	0 (0.0%)	0 (0.0%)	18 (27.3%)
Neutropenia	6 (9.1%)	3 (4.5%)	3 (4.5%)	12 (18.2%)
Decreased thyroid hormones	9 (13.6%)	0 (0.0%)	0 (0.0%)	9 (13.6%)
Leukopenia	6 (9.1%)	0 (0.0%)	0 (0.0%)	6 (9.1%)
Hyperglycemia	6 (9.1%)	0 (0.0%)	0 (0.0%)	6 (9.1%)
Increseased ALT level	6 (9.1%)	0 (0.0%)	0 (0.0%)	6 (9.1%)
Hypothyroidism	6 (9.1%)	0 (0.0%)	0 (0.0%)	6 (9.1%)
Increased uric acid	6 (9.1%)	0 (0.0%)	0 (0.0%)	6 (9.1%)
Increased AST level	6 (9.1%)	0 (0.0%)	0 (0.0%)	6 (9.1%)
Weight loss	6 (9.1%)	0 (0.0%)	0 (0.0%)	6 (9.1%)
Abnormal cardiac conduction	9 (13.6%)	0 (0.0%)	0 (0.0%)	9 (13.6%)
γ-GGT rise	3 (4.5%)	0 (0.0%)	0 (0.0%)	3 (4.5%)
Pneumonia	3 (4.5%)	0 (0.0%)	0 (0.0%)	3 (4.5%)
Hyponatremia	3 (4.5%)	0 (0.0%)	0 (0.0%)	3 (4.5%)
Abnormal liver function	3 (4.5%)	3 (4.5%)	3 (4.5%)	9 (13.6%)
Increseased creatinine level	3 (4.5%)	0 (0.0%)	0 (0.0%)	3 (4.5%)
Gastric reflux	3 (4.5%)	0 (0.0%)	0 (0.0%)	3 (4.5%)
Hypertension	3 (4.5%)	0 (0.0%)	0 (0.0%)	3 (4.5%)

CTCAE: Common Terminology Criteria Adverse Event; ALT: alanine transaminase; AST: aspartate transaminase; γ-GGT: γ-glutamyltransferase.

#### 2.1.2 化疗相关AEs

排除免疫相关AEs，与化疗相关AEs共发生156次，其中1级-2级AEs为150次，3级-4级AEs为6次。最常见AEs也是骨髓抑制：包括中性粒细胞减少27例（40.9%）、血小板减少27例（40.9%）、贫血24例（36.4%）、白细胞计数减少24例（36.4%）（表3）。

**表3:** 化疗相关不良事件

Adverse events	CTCAE 5.0 grade			Total
	Grade 1-2	Grade 3	Grade 4	
Neutropenia	24 (36.4%)	3 (4.5%)	0 (0.0%)	27 (40.9%)
Thrombocytopenia	27 (40.9%)	0 (0.0%)	0 (0.0%)	27 (40.9%)
Anemia	24 (36.4%)	0 (0.0%)	0 (0.0%)	24 (36.4%)
Leukopenia	21 (31.8%)	3 (4.5%)	0 (0.0%)	24 (36.4%)
Hair loss	9 (13.6%)	0 (0.0%)	0 (0.0%)	9 (13.6%)
Weight loss	6 (9.1%)	0 (0.0%)	0 (0.0%)	6 (9.1%)
Increased uric acid	6 (9.1%)	0 (0.0%)	0 (0.0%)	6 (9.1%)
Hypothyroidism	6 (9.1%)	0 (0.0%)	0 (0.0%)	6 (9.1%)
Hypertension	3 (4.5%)	0 (0.0%)	0 (0.0%)	3 (4.5%)
Increased creatinine level	3 (4.5%)	0 (0.0%)	0 (0.0%)	3 (4.5%)
Hyperglycemia	3 (4.5%)	0 (0.0%)	0 (0.0%)	3 (4.5%)
Abnormal liver function	3 (4.5%)	0 (0.0%)	0 (0.0%)	3 (4.5%)
Abnormal cardiac conduction	9 (13.6%)	0 (0.0%)	0 (0.0%)	9 (13.6%)
Increased ALT level	3 (4.5%)	0 (0.0%)	0 (0.0%)	3 (4.5%)
Decreased thyroid hormones level	3 (4.5%)	0 (0.0%)	0 (0.0%)	3 (4.5%)

### 2.2 新辅助治疗对患者生活质量的影响

通过EORTC评价NSCLC患者QoL-C30生活质量问卷，比较患者术前3周与术后5个周期的问卷调查结果，患者第1次和第2次QoL-C30评分分别为（47.16±6.55）分和（43.43±7.00）分，第4次和第6次分别为（42.76±5.54）分和（40.78±4.38）分，第7次和第8次分别为（40.63±4.38）分和（40.45±4.35）分，提示随着时间的推移，患者生活质量指标明显改善，差异具有统计学意义（P<0.05），见图1。

**图1:**
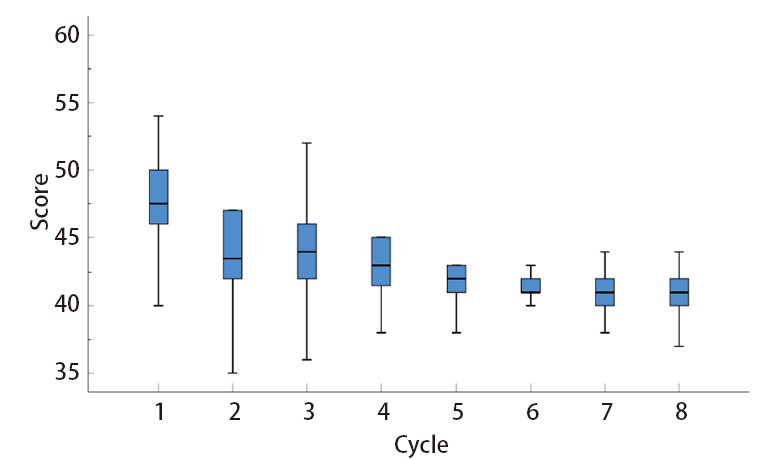
各周期生活质量评价得分箱图

## 3 讨论

流行病学调查研究^[16]^显示，随着居住环境的改变以及人们饮食结构的变化和生活节奏的加快，肺癌发病率一直居高不下，其中主要以NSCLC为主。NSCLC患者预后较差，5年生存率在15%以下。NSCLC常见表现为咳嗽、咯血、哮鸣、胸痛、发热，若病灶出现在较大支气管，会诱发刺激性咳嗽，随着疾病进展对支气管引流产生影响，引起感染。早中期NSCLC患者可手术根治，晚期NSCLC患者肿瘤病灶大，已侵犯局部组织或部分发生转移，临床往往通过射频消融、化疗、放疗等方案，以缩小病灶、遏制肿瘤扩散，进而延长患者生存期^[17]^。

作为一种系统治疗方法，新辅助治疗适用于未发生远处转移或处于进展期的恶性肿瘤，属于肿瘤细胞减灭治疗方法，术前给予患者新辅助治疗，可有效杀灭肿瘤细胞，使肿瘤负荷显著下降，降低病理分期，降低手术难度，提高病灶切除率^[18]^。在治疗过程中，AEs和SAEs是药物临床试验中重要的安全性指标，本研究结果显示，66例患者经过治疗总体安全性良好，免疫相关AEs和化疗相关性AEs共发生300次，其中1级-2级AEs 282次，3级-4级AEs 18次，其中SAEs 6次。结果提示患者接受新辅助治疗获益的同时，也不可避免会出现AEs，但总体安全性好。本研究中，AEs和SAEs发生率均低于郝娜等^[19]^报道，说明通过观察与监控，及时发现问题、解决问题，可显著降低治疗相关AEs。常见AEs为骨髓抑制，通过对症处理可耐受，其余AEs如胃肠反应、高血压、肝功能异常等通过对症处理，CTCAE 5.0 3级-4级患者通过减量可完成余下疗程。目前关于新辅助治疗所致AEs机制尚未完全明确，考虑AEs可通过自身反应性T细胞、细胞因子等多种不同途径而发生。AEs与患者全身多个脏器相关，根据受累靶器官的不同，其表现及分级也呈现出一定的差异性。通常1级-2级AEs症状较轻，无需住院接受治疗；3级AEs有明显的症状，需要遵照医嘱接受药物治疗，以防持续加重；4级AEs患者往往体征不稳定，生命安全受到威胁，需要收入重症监护室接受进一步治疗。因此在试验过程中，医护人员应密切关注受试者AEs和SAEs发生的情况，及时与研究者进行沟通，做到有效防范和及时处理，防止受试者AEs进一步发展，做到及时、准确、规范地填写记录，记录AEs出现的时间及持续时间，按照相关标准评估其严重程度，并积极采取有效的干预措施，以促进患者疾病转归，保障临床试验受试者生命安全。

辅助治疗对患者生活质量的影响是本研究观察的另一个指标。本研究结果显示，通过监管AEs，患者生活质量明显改善，且随着时间推移，QoL-C30评分逐渐下降，由新辅助治疗前的（47.16±6.55）分下降到新辅助治疗后的（40.45±4.35）分，可能原因为：治疗起效的同时，患者肿瘤负荷下降，由肿瘤引起的临床症状减轻，治疗获益，生活质量提高，治疗依从性提高。

作为药品管理部门，应明确自身的职责，高度重视临床试验期间AEs的监测和监管工作，通过建立并完善风险防范制度、应急预案，并加强医护人员对AEs评判和处理的专业培训，以增强其安全意识，从源头上控制风险，为患者提供优质的医疗服务；并通过加强患者及家属的健康教育，尽可能消除患者治疗中的安全隐患和差错。近年来，医学界关于恶性肿瘤免疫治疗的AEs认识不断加强，围手术期免疫相关AEs受到了高度关注，同时临床也对AEs管理提出了更高的要求。现有的循证医学证据尚无法满足临床的需求，胸部外科医生、肺癌诊疗专家面临知识结构重组及专业延展，因此需要进一步完善肺癌免疫治疗相关AEs的诊疗理念，以期为临床提供可靠的依据与有效的指导。

综上所述，临床试验工作中实施AEs的监管能够有效减少AEs，对确保新辅助治疗顺利进行具有重要意义，应予以高度关注。

**Competing interests**

The authors declare that they have no competing interests.
